# Does a PEEK Femoral TKA Implant Preserve Intact Femoral Surface Strains Compared With CoCr? A Preliminary Laboratory Study

**DOI:** 10.1007/s11999-016-4801-8

**Published:** 2016-03-28

**Authors:** Kathryn E. Rankin, Alexander S. Dickinson, Adam Briscoe, Martin Browne

**Affiliations:** 1 Bioengineering Science Research Group, Faculty of Engineering and the Environment, University of Southampton, Southampton, Hants SO17 1BJ UK; 2 Invibio Ltd, Thornton Cleveleys, UK

## Abstract

**Background:**

Both the material and geometry of a total knee arthroplasty (TKA) component influence the induced periprosthetic bone strain field. Strain, a measure of the local relative deformation in a structure, corresponds to the mechanical stimulus that governs bone remodeling and is therefore a useful in vitro biomechanical measure for assessing the response of bone to new implant designs and materials. A polyetheretherketone (PEEK) femoral implant has the potential to promote bone strains closer to that of natural bone as a result of its low elastic modulus compared with cobalt-chromium (CoCr).

**Questions/purposes:**

In the present study, we used a Digital Image Correlation (DIC) technique to answer the following question: Does a PEEK TKA femoral component induce a more physiologically normal bone strain distribution than a CoCr component? To achieve this, a DIC test protocol was developed for periprosthetic bone strain assessment using an analog model; the protocol aimed to minimize errors in strain assessment through the selection of appropriate analysis parameters.

**Methods:**

Three synthetic bone femurs were used in this experiment. One was implanted with a CoCr femoral component and one with a PEEK femoral component. The third (unimplanted) femur was intact and used as the physiological reference (control) model. All models were subjected to standing loads on the corresponding polyethylene (ultrahigh-molecular-weight polyethylene) tibial component, and speckle image data were acquired for surface strain analysis using DIC in six repeat tests. The strain in 16 regions of interest on the lateral surface of each of the implanted bone models was plotted for comparison with the corresponding strains in the intact case. A Wilcoxon signed-rank test was used to test for difference at the 5% significance level.

**Results:**

Surface analog bone strain after CoCr implantation indicated strain shielding (R^2^ = 0.6178 with slope, β = 0.4314) and was lower than the intact case (p = 0.014). The strain after implantation with the PEEK implant deviated less from the intact case (R^2^ = 0.7972 with slope β = 0.939) with no difference (p = 0.231).

**Conclusions:**

The strain shielding observed with the contemporary CoCr implant, consistent with clinical bone mineral density change data reported by others, may be reduced by using a PEEK implant.

**Clinical Relevance:**

This bone analog in vitro study suggests that a PEEK femoral component could transfer more physiologically normal bone strains with a potentially reduced stress shielding effect, which may improve long-term bone preservation. Additional studies including paired cadaver tests are necessary to test the hypothesis further.

## Introduction

Loss of distal femoral bone mineral density (BMD) is commonly reported after TKA [9, 10, 19, 22, 23, 29]. Strain, a measure of the local relative deformation in a structure, corresponds to the mechanical stimulus that governs bone remodeling [7] and is therefore a useful in vitro biomechanical measure for assessing the response of bone to new implant designs and materials [30]. Periprosthetic bone remodeling may be attributed to local changes in the mechanical strain field of the bone (ie, the distribution of strains on and within the bone) as a result of the stiffness mismatch between high-modulus metallic implant materials and the supporting bone, which leads to stress shielding [8, 13]. Substantial loss of periprosthetic BMD may promote implant loosening and complicate revision surgery. Implantation in inadequate bone stock remains one of the most difficult tasks surgeons face at revision; therefore, minimizing the stress shielding effect of TKA implants could be of great value to both the patient and the surgeon [17, 33].

There are established theories that bone’s mechanical adaptation stimulus is related to the strain it experiences [7]. Numerical modeling and in vitro experimental work has indicated general correspondence between the change in the bone strain field after implantation and the progressive remodeling changes observed in clinical measurements [3, 5, 27, 32]. Digital image correlation (DIC) is a noncontact image analysis technique increasingly used in biomechanics for full-field strain assessment of complex three-dimensional surface geometry, including heterogeneous, anisotropic materials such as bone tissue [24]. The full-field nature of DIC permits measurements at multiple regions of interest to make specific strain comparisons for the evaluation of initial bone adaptation stimulus. As detailed by Sutton et al. [24], the local image correlation algorithm tracks the displacement of a random speckle pattern within a specified analysis area by matching smaller subset areas of unique gray-level pixel values, spaced center to center by a specified “step size” (in pixels), between images obtained before and after deformation. Hence, the subset size (in pixels) defines the spatial resolution of the displacement measurement. The strain is calculated from the grid of displacement data points to form a Green-Lagrange strain tensor for each point in the grid. These strain tensors are then smoothed over a specified decay “filter size” or “strain window” (of a number of data points) to reduce noise, which therefore controls the spatial resolution of the strain measurement ([filter size × step size] + subset size). Optimization of the DIC parameter selection is necessary to produce valid results and minimize noise, bias, and systematic errors during data analysis [4, 31, 34]. Studies should explicitly state the procedure involved in their analysis to ensure reliability of the results and reproducibility between studies. However, this is uncommon in the documentation of biomechanical studies [6, 15, 16, 20, 25, 26, 28].

The reduced stiffness of polyetheretherketone (PEEK) implants (4 GPa) compared with cobalt-chromium (CoCr) implants (220 GPa) has the potential to reduce stress shielding, as evidenced by the review of Kurtz and Devine [11]. At the time of writing, no published studies were found that had investigated this for TKA. Therefore, in the present study, we used a DIC technique to answer the following question: Does a PEEK TKA femoral component induce a more physiologically normal bone strain distribution than a CoCr component? To this end, a standardized procedure for DIC analysis parameter verification is developed and presented for the evaluation of implanted constructs.

## Materials and Methods

### Test Specimens

Three medium anatomical foam femur models (Sawbones Europe AB, Malmö, Sweden) were sectioned and potted in Technovit® acrylic resin (Heraeus Medical GmbH, Wehrheim, Germany). These closed-cell polyurethane foam models have realistic geometry generated from CT data and comparable microstructure and nonlinear stress-strain characteristics to cancellous bone [21]. The samples were aligned to represent stance such that the anatomical axis was 6° adducted from the mechanical axis and 3° to the vertical axis [18]. One distal femur was implanted with a size C metallic (CoCr) femoral TKA component (*E* = 220 GPa) and another femur was implanted with a PEEK-OPTIMA^®^ (PEEK) (Invibio Ltd, Thornton Cleveleys, UK) implant (*E* = 4 GPa) of the same size and geometry (Freedom Knee^®^; Maxx Orthopaedics Inc, Plymouth Meeting, PA, USA) machined from extruded stock. Both components were fixed using Palacos R acrylic bone cement (Heraeus Medical GmbH) mixed under vacuum. One femur was left intact for reference as the nominal physiological strain case.

**Fig. 1:**
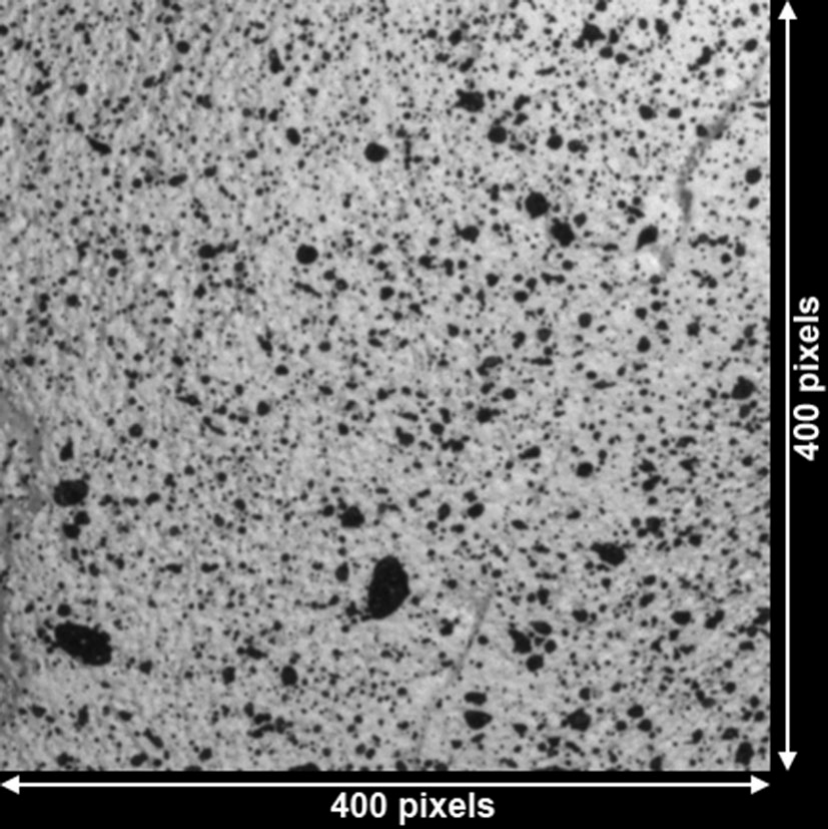
The speckle pattern size ranged from 2 to 30 pixels across the analog bone surfaces.

To facilitate surface strain measurement, each femur was sprayed with a thin layer of matte white paint followed by a black acrylic paint speckle pattern applied using a brush-flicking technique. This resulted in a speckle pattern coverage of approximately 22% (estimated by converting images to binary to assess speckle size and coverage [12]). The speckle diameter ranged from 2 to 30 pixels (Fig. 1).

### DIC Test Technique

Dual 2 megapixel cameras (1624 × 1232 pixels) (Limess GmbH, Krefeld, Germany) were used to acquire speckle image data on the lateral bone surface with an exposure time of 12 ms and an aperture of f12 after calibration with a 12 × 9 grid of 5-mm targets. The cameras were positioned with a relative pan angle of 25°, a baseline of 139 mm, and a focal length of 308 mm, resulting in a spatial image resolution of approximately 40 µm/pixel. Three fiberoptic light sources were used to illuminate the anterior, lateral, and posterior bone surfaces with a diffuse LED light source positioned behind the cameras, ensuring that there was no pixel saturation (0-255 gray-scale values) that could cause measurement uncertainty.

Each distal femur was loaded to 750 N against the corresponding all-polymer ultrahigh-molecular-weight polyethylene tibial component and a planar bearing using an Instron 5569 electromechanical test machine (Instron Inc, Norwood, MA, USA) to achieve an optimal DIC signal-to-noise ratio. The planar bearing allowed x and y sliding motion such that the specimen could deflect to help ensure compressive loading. Six repeat tests were carried out to assess experimental error in measurement of surface strain under loaded conditions and to account for variation in tibial component positioning.

In each test, a ramped displacement was applied until the target load was attained, at which point the crosshead was held at a constant displacement while six consecutive images were taken (2 fps) to assess measurement variability resulting from sensor noise. Pre- and posttest static images were obtained for each bone model under unloaded nominally zero strain conditions to quantify the displacement and strain resolutions. In addition, a vertical rigid body translation was carried out, where the specimen was moved 2.5 mm vertically and images taken before and after the movement. This assessed the software’s ability to perform a rigid body correction, recognizing that the speckle pattern had moved, yet not deformed (and correct for this), to determine any effect on the resulting strain resolution.

### Displacement and Strain Calculation

**Fig. 2:**
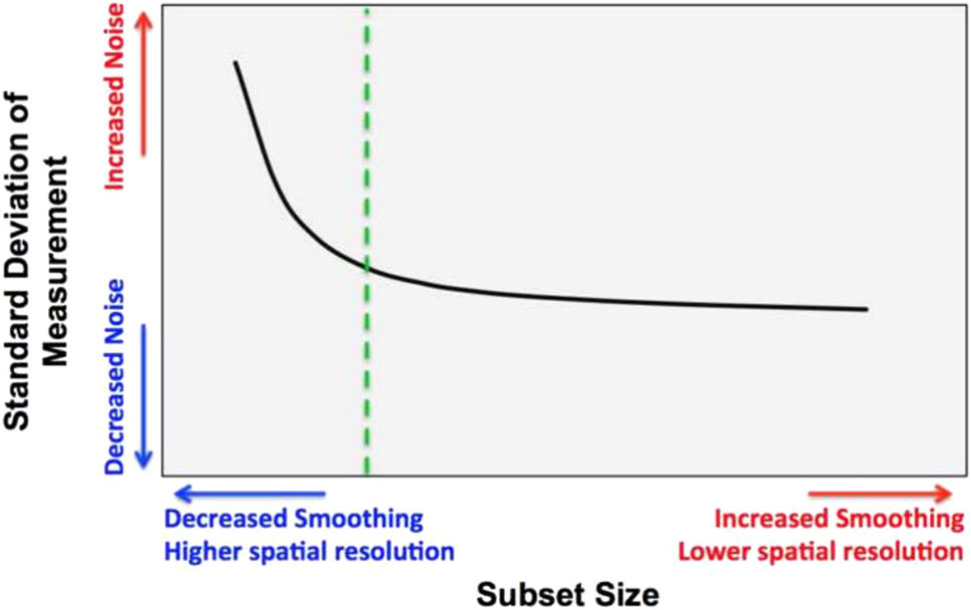
The selection of DIC subset size is a tradeoff between noise and smoothing of the data under nominally zero strain conditions (an example curve is shown where the green dashed line represents the subset size that may be optimal).

**Fig. 3:**
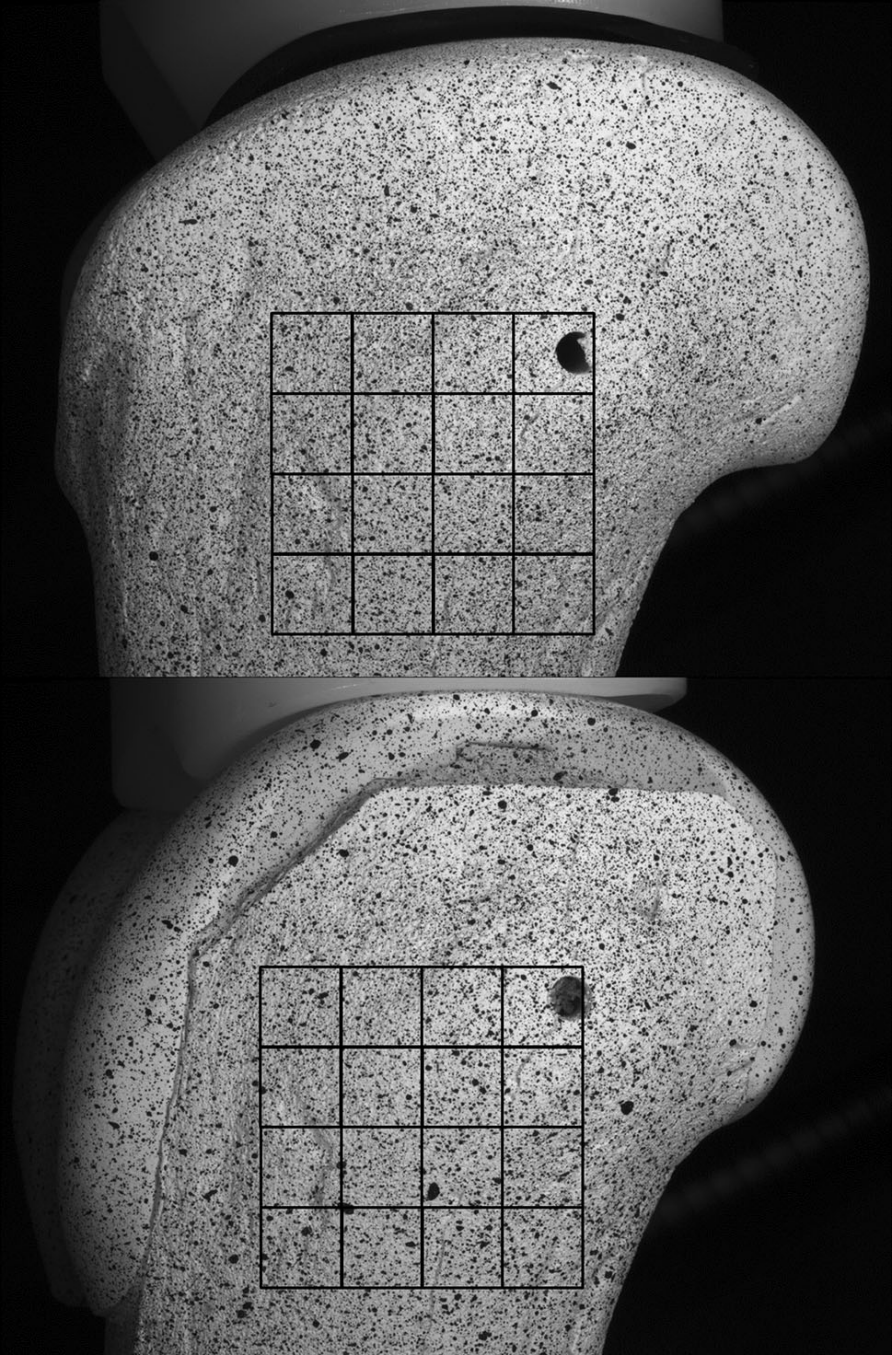
The virtual strain gauge ROIs were positioned on the lateral surfaces of the intact and implanted bone models for comparison of strain measurements.

To test the hypothesis that a PEEK femoral component will induce more physiologically normal strains compared with CoCr, the speckle images obtained in the experiment were analyzed using Vic-3D software (Correlated Solutions Inc, Columbia, SC, USA) to calculate the displacement and thus the strain fields (the primary study outcome variable) under loading. To determine the optimum DIC parameters (subset and step size) in terms of maximum strain gradient sensitivity versus noise (Fig. 2), the SD of horizontal and vertical displacements (*U* and *V,* respectively) were evaluated, under unloaded nominally zero strain conditions, both static and after rigid body translation correction. Using the same conditions, a suitable decay filter size for the computation of the Green-Lagrangian strain field was determined from the SD of strain across a range of subset and step sizes. The mean and SDs of displacement and strain under nominally zero strain conditions were taken as the respective measurement bias and resolution. The raw image noise was assessed by comparing the SD of pixel difference between consecutive unloaded images. The first and second principal strain measurements under load were averaged within 16 5-mm^2^ virtual strain gauge regions of interest (ROIs) across the lateral side of each bone model for quantitative comparison of the tensile and compressive surface strains, respectively (Fig. 3). The measurement variability was calculated from the SD of the strain measurements in each repeat test across the ROIs. The ROI principal strain values were plotted for the intact versus implanted cases, and the regression score (R^2^) and gradient (β) were calculated using IBM SPSS Statistics 20 (IBM Corp, Armonk, NY, USA). Perfect agreement between the implanted and intact cases would give R^2^ = 1 and β = 1. A Wilcoxon signed-rank test was used to test the null hypothesis that there would be no difference between the strain at relative ROI locations on the intact and implanted bone model surfaces at the 5% significance level.

### DIC Parameter Selection

Evaluation of a gray-scale pixel difference from one consecutive static raw image to the next gave a maximum SD of 2.846 (range, 0-255) in the implanted PEEK speckle images, corresponding to 1% raw image noise value. The highest displacement and strain SDs under static nominally zero strain conditions were also measured for the implanted PEEK case. Hence, the DIC analysis parameters were determined from this test specimen and the consequent measurement resolutions are presented as the worst case for the DIC technique used.

**Fig. 4:**
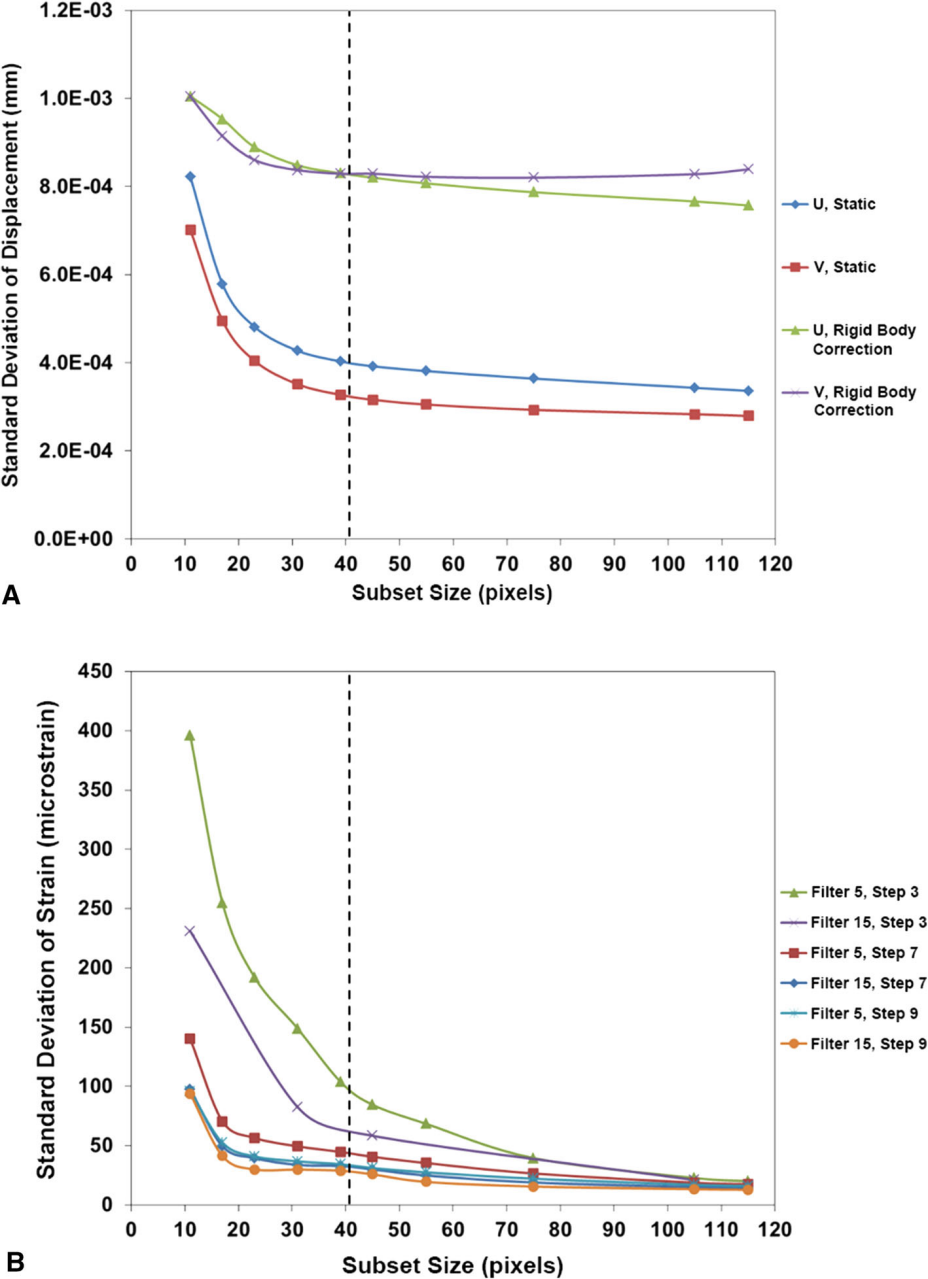
**A**–**B** (**A**) The variation in SD of horizontal and vertical displacements, U and V, respectively, with subset size indicated that 41 × 41 pixels was optimal for the DIC analysis from both the static (unloaded zero strain state) and rigid body correction noise analyses. (**B**) The variation in SD of strain with subset size (in pixels), step size (step, in pixels), and strain filter size (in data points) is shown for comparison.

The SD of displacement and strain under static zero strain conditions with increasing subset size was plotted to aid analysis parameter selection (Figs. 4A and 4B, respectively). A subset size of 41 × 41 pixels was chosen as a balance between noise and smoothing of the displacement field while providing a large amount of unique data (speckle diameter range 2-30 pixels). A step size of 7 pixels and a filter size of 15 data points were chosen using the same criteria for the strain field across the bone model surface (resulting in > 16,000 data points).

### DIC Resolution

With the selected subset and step sizes, under static conditions, the maximum displacement resolution (SD) was 0.008924 pixels (0.36 µm) with a mean of -0.00635 pixels (-0.25 µm). The maximum strain resolution after filtering was a SD of 30 µε with a mean of 38 µε. After the translation test under nominally zero strain conditions, the rigid body correction performed by the software gave a maximum strain resolution SD of 46 µε with a mean of 74 µε.

### Experimental Error

The experimental error of strain measurement was ± 71 µε, ± 33 µε, and ± 24 µε for the intact, PEEK, and CoCr implanted bone models, respectively. This gave a maximum six-sigma experimental error (representing 99.7% spread of data error) of ± 213 µε or 9.7% of the maximum strain, 2200 µε.

## Results

**Fig. 5:**
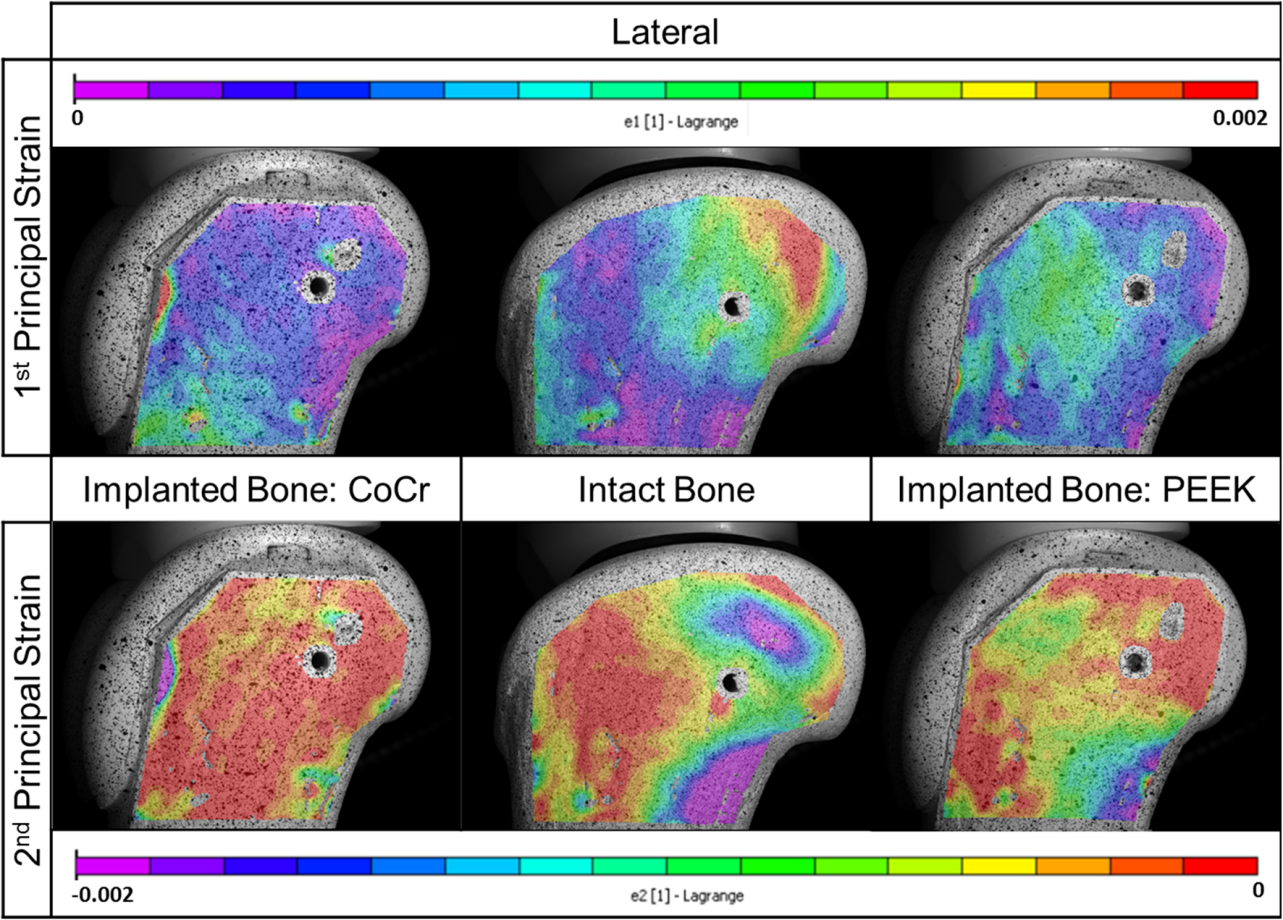
Principal strain maps are shown for the intact and implanted test specimens in the lateral view (first principal is tensile strain and second principal is compressive strain).

**Fig. 6:**
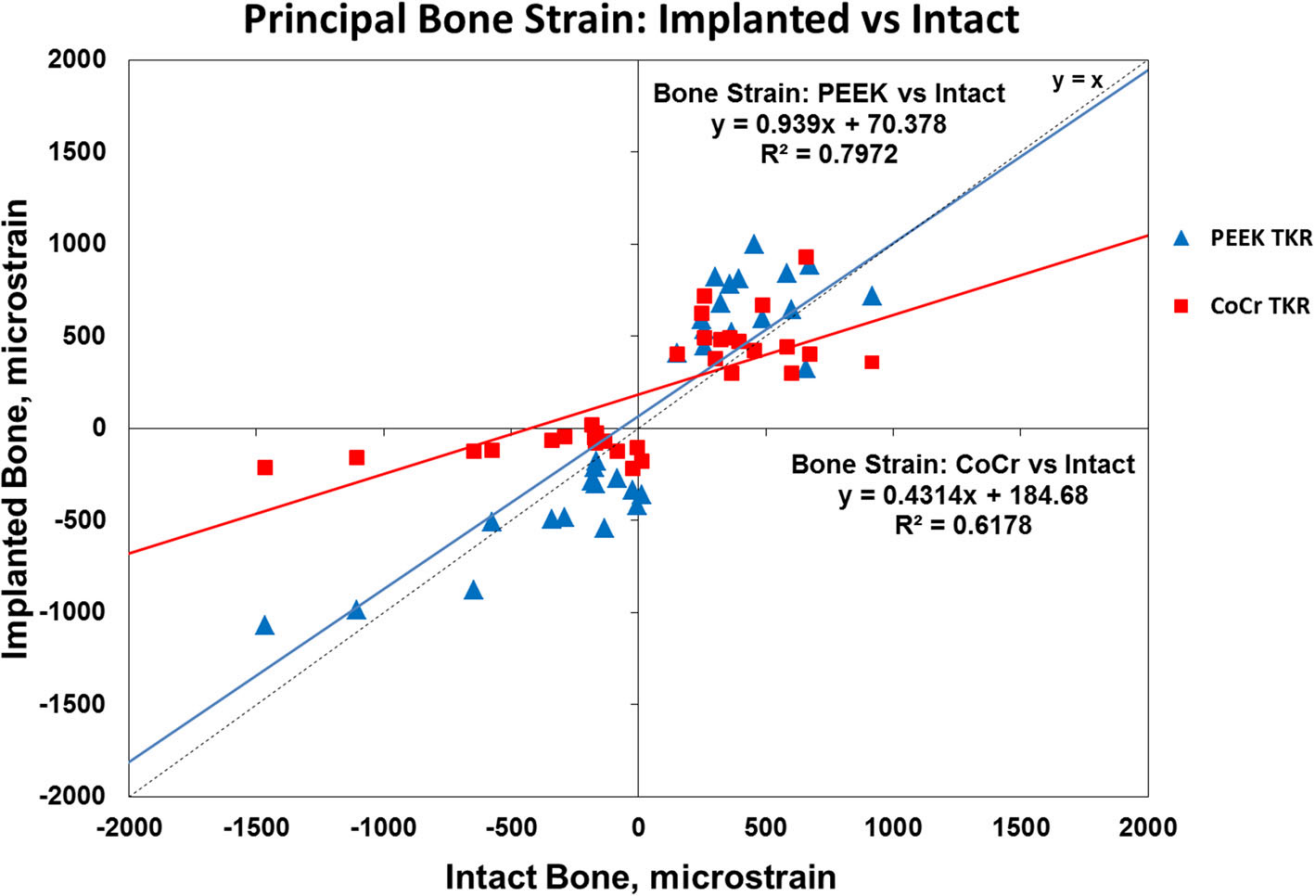
A quantitative comparison of principal strain on the intact analog bone surface with corresponding ROIs on the implanted cases showed that there was a large deviation with the CoCr bone model strain compared with the PEEK bone model strain.

The principal strain maps show the qualitative difference in surface strain distribution for the three cases; comparatively lower strains were measured on the model with the CoCr implant relative to the intact case, whereas the PEEK implant induced a strain distribution closer to the intact case (Fig. 5). Quantitatively, a larger deviation was observed between the CoCr-implanted bone model and the intact bone model data sets (R^2^ = 0.6178, slope β = 0.4314) with different (lower) strain measurements at the 5% significance level (p = 0.014) when analyzed using the Wilcoxon signed-rank test (Fig. 6). A closer agreement was found between the strain distribution of the PEEK and the intact data sets (R^2^ = 0.7972 with slope β = 0.939) with no difference (p = 0.231).

## Discussion

Loss of BMD may result from the stress shielding effect of TKA implants, which could lead to implant loosening and complex revision surgery [8, 17, 30]. The risk of aseptic loosening may be lowered, and the preserved periprosthetic bone stock may be improved by reducing the stress shielding effect of implant designs. The reduced stiffness of PEEK implants (4 GPa) compared with CoCr has the potential to reduce stress shielding, and this had not previously been investigated for TKA femoral components. The purpose of this study was to develop a DIC method to test the hypothesis that a PEEK femoral component in a TKA implant will reduce strain shielding in comparison to a contemporary CoCr component. Using analog foam femur models, the study found that the CoCr femoral component induced lower surface strain in comparison with the intact case, whereas the PEEK implant induced a surface strain distribution similar to that of the intact condition.

It should be noted that the results from this study are only comparative with one another as a result of the use of analog femur models and a single load case. Analog materials may not respond in a true physiological manner, because they do not represent real bone biological processes, microdamage, or soft tissue interactions. Furthermore, cancellous bone structural anisotropy is also not captured in these analog materials. However, the geometry and material properties of the analog femora are nominally the same and therefore direct comparisons are possible. Analog samples are also relatively inexpensive and easy to source. In contrast, cadaveric models are expensive and have high inter- and intrapatient variability, which may lead to greater disparity in the induced strain fields. In the absence of well-matched right and left paired bone samples, a cadaver study would require a large population for statistical analysis.

An additional limitation of this study is the use of one modeled implanted case for each type of femoral component. During implantation, the dimensions of the resected femur may have varied as a result of tolerances between the oscillating saw blade and the surgical cutting guide, which could have altered the thickness of the cement mantle and the induced surface strain distribution. These are, however, unavoidable design limitations of the implant system’s instrumentation used and are clinically representative. The spatial resolution of the strain field obtained using DIC is limited by the maximum size and minimum spacing density of the speckle pattern used (maximum speckle diameter 30 pixels and approximate speckle coverage of 22%); hence, a subset size in excess of 30 pixels was required in the DIC analysis to track the deformation of the pattern on the bone model surface without leaving gaps in the data, as similarly reported by Carriero et al. [2]. However, the geometry (low curvature) and material of the distal femur surface was such that there were no severe strain gradients that would require small subsets for accurate measurement, and the DIC parameters used were considered sufficient for this application [12].

Lower strains were measured across the surface of the bone model implanted with the CoCr femoral component compared with the intact reference model, whereas the cortex strain of the bone model implanted with the PEEK femoral component was not different from the intact reference model. This supports the hypothesis that a more compliant PEEK implant could promote a more physiologically normal strain distribution compared with that induced by a contemporary CoCr metallic implant, thus indicating the potential for successful long-term bone maintenance. The increase in strain in the anterior region of both implanted cases compared with the intact case may be caused by the change in geometry of the articular surface, causing anterior translation of the tibiofemoral contact. Relatively higher strain in regions close to the CoCr implant can be attributed to the high stiffness mismatch between the implant and bone model where the bone is constrained to the implant by the cement. As a result of the implant geometry, bone material in the central metaphyseal region of the femur is not so constrained by the CoCr implant and subsequently much lower compressive strains are observed compared with the intact bone model. This suggests that stress shielding could occur and may lead to bone resorption in vivo if the local strains are below the modeling threshold strain criterion for bone maintenance [7].

These findings are consistent with clinical measurements of longitudinal BMD changes around metallic TKAs, which have reported a reduction in density, particularly in the central metaphyseal region, attributed to stress shielding and reduced patient activity after surgery [1, 9, 22]. The experimental measurement of distal femoral bone strain after implantation of a TKA prosthesis has not been previously investigated to the authors’ knowledge. Importantly, this study has presented a selection process for the key DIC analysis parameters, which govern the reliability of implanted bone strain measurement. Previous knee implant studies using DIC to assess bone strain have focused on the tibial bone surface [14, 15, 20]. A DIC study on periprosthetic bone strains with postmortem retrieved TKA tibial components carried out by Mann et al. [15] suggested that a reduction in BMD leads to higher bone strains, which could lead to an increased risk of failure. In a study of the effect of unicompartmental knee implant design on proximal tibial strain in a Sawbone model, Scott et al. [20] also reported that metal-backed implants induced strain shielding compared with an all-polyethylene design. However, it was also concluded that the all-polymer design was associated with bone damage at the microscopic level as a result of its compliance and it was advised that these devices should be used with caution in patients likely to induce high loads. This requires further investigation for the PEEK femoral component using a cadaveric model, which would enable more realistic damage processes to be followed. In addition, the design and material parameters differ considerably for the unicompartmental component. It should also be noted that the wear, fixation, and structural integrity require investigation for a novel PEEK femoral component, but this is outside the remit of the present study.

In conclusion, the present study described a DIC methodology to evaluate femoral periprosthetic bone strain changes induced by CoCr and PEEK TKA implants in an analog bone model. The stress shielding effect predicted with the CoCr implant resulting from reduced surface strains occurred in regions similar to those reported with reductions in BMD after TKA [1, 9, 22], supporting the utility of the evaluation method. The PEEK implant produced a bone surface strain field closer to that of the intact bone case, suggesting that a PEEK femoral component could transfer more physiologically normal bone strains with a reduced stress shielding effect, potentially improving long-term bone preservation. Having established a method for assessment of implanted constructs in this preliminary study, further work will focus on application of DIC to a cadaveric model for preclinical assessment of TKA devices.
